# The right of women in property sharing in Bangladesh: Can the islamic inheritance system eliminate discrimination?

**DOI:** 10.1186/s40064-016-3347-2

**Published:** 2016-10-03

**Authors:** Issa Khan, Md. Faruk Abdullah, Noor Naemah Abdul Rahman, Mohd Roslan Bin Mohd Nor, Mohd Yakub Zulkifli Bin Mohd Yusoff

**Affiliations:** 1 Director Office, Academy of Islamic Studies, University of Malaya, 50603 Kuala Lumpur, Malaysia; 2 Department of Shariah and Economics, Academy of Islamic Studies, University of Malaya, 50603 Kuala Lumpur, Malaysia; 3 Department of Fiqh and Usul al-Fiqh, Academy of Islamic Studies, University of Malaya, 50603 Kuala Lumpur, Malaysia; 4 Department of Islamic History and Civilisation, Academy of Islamic Studies, University of Malaya, 50603 Kuala Lumpur, Malaysia; 5 Academy of Islamic Studies, University of Malaya, 50603 Kuala Lumpur, Malaysia

**Keywords:** Woman, Property sharing, Social practices, Islamic solution

## Abstract

This study seeks to discover the best solution for women’s property sharing between Islamic and current social practices in Bangladesh. A qualitative method has been adopted to achieve this goal. It is found that the majority of the women are marginalised from their property in the social practice. On the other hand, in the Islamic solution, the property is fixed for all classes of women and is based on a property sharing system called *fara’id* that takes into account the roles and responsibilities of man and woman in the society. Men are responsible for providing maintenance to their wives and children. Men in Islamic society should bear expenditure related to marriage. The research concludes that the Islamic solution is fair and ensures just property sharing rights for women. It suggests that the Islamic solution for property sharing should be implemented to empower women in Bangladesh.

## Background

Historically, gender plays a key role in the distribution of inheritance property (Kameri-Mbote 2002; Saleh 1972; Abdullah et al. 2014). In some jurisdictions, males are awarded the major share of the property. As an inheritor, a son and a daughter do not always share the same proportion of the property (Ahmed 2007). However, in some jurisdictions, males and females are always equal in their share of the inherited property (Mojahid 1989, p. 268). In some legal systems, including in India, women are deprived of any share in the property (Green 2015).

Equality between men and women in the distribution of inheritance has become a major concern (Shatzmiller 1995). According to the current Western ideology, a woman should have equal right to the inheritance. It is claimed that the property distribution in Islam is not fair (Barlow and Akbarzadeh 2006). According to Islamic jurisprudence, a son will get a share which equals the share of two daughters (Quran, 4:11). The husband will get half of the deceased wife’s share if she does not have any child while the wife will get one-fourth of the deceased husband’s share if he does not have any child (Uthaimin 1983, p. 45; Bagovi 1999, vol. 3, p. 130). When the deceased has only one son, he is entitled to the whole property (Atiyah 2008, p. 137), but if the deceased left only one daughter then the maximum she is entitled to have a half of the property, and the remainder will be distributed among the brothers and sisters of the deceased (pp. 92–95). Based on this, many claims that woman’s property sharing in Islamic Shari’ah is not equal to man (Steinzor 2003).

However, Muslim scholars argue that the property right of the woman in Islam is fair (Labib 1995, p. 7; Al-Tabari 2000, vol. 8, p. 2 70). The property distribution is made based on the role and responsibility of man and woman in the Muslim society (Al-Qurtubi 1964, vol. 5, p. 168). In the Islamic social system, a man has more responsibility than a woman (Dangor 2001). A man is responsible for providing *nafaqah* (money that the husband has to provide to the wife for her sustenance) to his wife and children (Al-Tabari 2000, vol. 8, p. 270). Moreover, Muslim scholars point out that Islamic inheritance distribution is comprehensive (Barlow and Akbarzadeh 2006). An examination of the various cases of distribution of inheritance property reveals several cases where man and woman get equal shares (Quran, 4:11; Al-Dawlibi 1983).

Bangladesh is a 90 % Muslim populated country which until recently followed Islamic Shari’ah rules relating to inheritance (Social Institutions & Gender Index 2014; Abdullah et al. 2015). Recently, the government of Bangladesh has attempted to revise this law by legislating equal property rights between man and woman relating to inheritance. This change of law was based on the government’s national women development policy (NWDP) (2011) aimed to provide an equal share to women in property and opportunities in work and business (The Daily Star, March 8, 2011). This led to a nationwide strike by the supporters of Shari’ah law against this new law (BBC News, April 3, 2011). Though the case is highlighted mostly as a political matter, an academic analysis is necessary to justify the legitimacy and fairness of the Islamic system of inheritance. It might be questioned that though a daughter’s share is a half of the son’s, what are the reasons for it to be considered just distribution. As mentioned earlier, the Shari’ah has a comprehensive inheritance distribution law. Therefore, we should review all the rules related to inheritance property. Each and every individual’s share of the inheritance need to be examined thoroughly.

This study analyses how women are being discriminated in the societal practice of property distribution in Bangladesh. It examines whether a new model of inheritance property distribution is necessary for women or the Islamic solution is sufficient. It will justify whether the Islamic system of inheritance gives women maximum share in property compared with the societal practice of property distribution in the country.

## Methodology of the study

This study adopts the qualitative method wherein collect relevant information is collected from articles, classical and modern books, newspapers, and websites. Along with this, the Holy *Quran, Sunnah* (the tradition of Prophet Mohammad) and *Tafsir* (the interpretation of the Holy Quran) are referred to in this research. The researchers compare both systems for women property sharing practices in Bangladeshi society and the Islamic property sharing system to determine which ensures more property right for women.

## Inheritance distribution system in Islamic law

Islamic inheritance law is called *al*-*faraid*. *Faraid* is constituted by the *holy Quran* and the *Sunnah* (Chowdhury 1964). It is an important part of Shari’ah. Encouraging Muslims to learn *faraid*, the Prophet Muhammad (PBUH) said, “Learn *faraid* and teach them to people, because it is one-half of knowledge and it will be forgotten and the first to be taken up from my community” (Al-Darqutni 1966, vol. 4, p. 67, Hadith no. 1).

*Faraid* is very comprehensive compared with other inheritance laws as it discusses the share of different types of heirs. A large body of classical Islamic literature on *faraid* thoroughly discusses the rules of property distribution among the heirs and solves a number mathematical problems. *Faraid* was also appreciated by some non-Muslims. Admiring *faraid* Rumsey (2009) mentioned, “The Muslim law of inheritance comprises beyond question, the most refined and elaborate system of rules for the devolution of property that is known to the civilised world.”

Several verses of the Holy Quran constitute the *faraid.* Firstly, the Quran mentions that there is a fixed share both for man and woman in the inheritance. Allah says, “From what is left by parents and those nearest related, there is a share for men and a share for women whether small or large—a fixed share” (Quran, 4:7). After that, Allah declares the share of man and woman in the following verse, “God enjoins you about your children that a boy’s share is equal to that of two girls. And, if there are only girls among the children and they are more than two, then they shall receive two-thirds of the inheritance, and, if there is only one girl, then her share is half” (Quran, 4:11). This verse is the root of *faraid* which decides the share of a son is equivalent to two daughters. A daughter will get half of the property if there is no son. If there is more than one daughter, then they share two-thirds of the property. Others will share the rest of the property. If there is only a son, then he is entitled to all the property (Uthaimin 1983, p. 45).

The *fariad* also mentions the parents’ share of the deceased’s property as the Quran mentions, “And if the deceased has children, then the parents shall inherit a sixth each, and if he has no children and only the parents are his heirs, then his mother shall receive a third, and if he has brothers and sisters, then the mother’s share is the same one-sixth after the payment of any legacies he may have bequeathed and after discharging any debts he may have left behind. You know not who among your children and parents are nearest to you in benefit. This is the law of God. Indeed, God is Wise and All-Knowing” (Quran, 4: 11).

The husband and wife’s shares are prescribed as, “And to you belongs a half of what your wives leave, if they die childless. Moreover, if they have children, a quarter of what they leave shall be yours after payment of any legacies they may have bequeathed and after discharging any outstanding debts. Your wives shall inherit a quarter of what you leave, if you die childless. If you have children, then they shall inherit one-eighth, after payment of any legacies you may have bequeathed, and after discharging any of your outstanding debts” (Quran 4: 12).

For the brother and sister’s share, the Quran mentions, “They request from you a legal ruling. Say, “Allah gives you a ruling concerning one having neither descendants nor ascendants as beneficiaries. “If a man dies, leaving no child but only a female sibling, she will have half of what he left. And he inherits from her if she dies and has no child. But if there are two female siblings or more, they will have two-thirds of what he left. If there are both male siblings and female siblings, the male will have the share of two females. Allah makes clear to you His law, lest you go astray. And Allah is knowing of all things” (Quran, 4: 176). This indicates that the brother can get all the property of the sister if she does not have any child and parents but the sister can get only half of the property if the brother dies and he does not have any child or parents.

The remaining portion of the inheritance will be given to the nearest male relative. The Prophet (PBUH) mentioned, “Give the heirs their share and if something remains, it is for the closest male relative” (Hajjaj 1972, vol. 5, p. 59, hadith no. 4226). Based on the verses of the Quran and the prophetic narrations, the types of heirs can be divided into two. Firstly, the heirs who have fixed portion of share on the total property. They are called ‘*ashabul furud’*. The example of *ashabul furud* is the mother and father (when the children of the deceased are alive), husband or wife. Secondly, the heirs who share the property after the heirs of the first category have received their share. They are called ‘*asaba*’. Son and father (when there are no children) represent the *asaba*. After *ashabul furud* have received their fixed shares, the *asaba* will get the rest of the property (Uthaimin 1983, p. 45). One person may belong to both of the above categories in different situations. For example, a father is considered *ashabul furud* when the deceased has children. When the deceased does not have children, then the father is considered as *asaba* and is entitled to the rest of the property.

A son is an important inheritor with a significant impact on property distribution. If the son is alive, then the brothers and sisters of the deceased will not get any share. When the son is alive, then the daughters will be *asaba*. If the son is absent, then the daughter will be *ashabul furud*. In the absence of children, the husband’s share will be one-half on the wife’s property, and when present it is one-fourth (Barraz 1981, p. 475). Similarly, the wife gets one-fourth if there is no children but one-eighth if there are children. Every inheritor’s share in different cases has been given below (Tables 1, 2).

**Table 1:** *Faraid* Mentioned in the Quran

Inheritor	Property owned by	Share of property
Daughter (one daughter and no son)	Father	1/2
Daughter (more than one and no son)	Father	2/3
Daughter (with son)	Father	Residuary, one daughter gets 1/2 of one son
Mother (If there is grandson)	Son	1/6
Mother (If there is no grandson)	Son	1/3
Wife (If there is no son)	Husband	1/4
Wife (If there is son)	Husband	1/8
Half-sister from the mother’s side (one only and there is no descendants and parents of the deceased)	Brother	1/6
Sister (more than one and there is no descendants and parents of the deceased)	Brother	All get 1/3 of the property
Uterine sister (one only and there is no descendants and parents of the deceased)	Brother	1/2
Uterine sister (more than one and there is no descendants and parents of the deceased)	Bother	1/3

*Sources*: Quran, 4: 11, 12,176

**Table 2:** *Faraid* is derived from the prophetic tradition (Sunnah), consensus of the Muslim scholars’ decisions (Ijma) and analogy (Qiyas)

Inheritor	Property owned by	Share of the property
Granddaughter (son’s daughter), If the daughter is not alive	Grandfather	1/2
Granddaughter (more than one)	Grandfather	2/3
Granddaughter with one daughter	Grandfather	1/6
Half-sister from father’s side (If there is no uterine sister)	Half-brother from father’s side	1/2
Half-sister from father’s side, more than one (If there is no uterine sister)	Half-brother from father’s side	2/3
Half-sister from father’s side with half-brother from father’s side	Half-brother from father’s side	Residuary, one sister gets ½ of one brother
Half-sister from father’s side with one daughter or son’s daughter	Half-brother from father’s side	Residuary, she will get what is left
Half-sister from father’s side with uterine sister	Half-brother from father’s side	1/6
Grandmother (both maternal & paternal), if there is no mother	Grandson	1/6

*Sources*: Al-Kardi (2010)

## Women status in Bangladesh

Bangladesh emerged as an independent country in 1971. Prior to that, it was under the Pakistani Government and was named East Pakistan (Alam et al. 2011). Since its independence, the government has been trying to promote the status of women and gender parity in education, employment, and income. The constitution of Bangladesh supports equal right for men and women (The Lawyers and Jurists 2012). Women have been taking part in the parliamentary election since 1973 (Hossain and Tisdell 2005). A series of laws have been passed to ensure women’s rights in Bangladesh. Research indicates that nowadays educational and wage gaps between males and females are reducing gradually (Koenig et al. 2003). However, some studies show that laws related to women’s rights remain ineffective because of culturally negative views toward women’s rights, lack of good governance, and some socioeconomic factors (Begum 2004, p. i).

## Property sharing in Bangladeshi society

Discrimination against women is an ongoing debate. Historically women are given less important compared to men (Mohammad 2013). In Bangladesh, women suffer various problems to achieve their right. The major issue is their unjust and limited right to explore resources (Sourav 2015). Social norms and culture frequently ignore women’s rights in Bangladesh (Abdullah et al. 2015). Women are more deprived than men in their access to financial assets, healthcare, and education. This includes Hindu and Christian women. Traditions and socio-cultural norms have marginalised women of Bangladesh from achieving their financial independence (Parveen 2007). Although inheritance practices in the society are supposed to accord to Shari’ah law, the socio-cultural values and norms have a strong influence on the issue of women inheritance in Bangladeshi society. Women are discriminated within their household (Sultana and Zulkefli 2012). Parents are happier with the birth of sons because as they think sons would help and provide financial support in the old age. On the other hand, daughters are considered a burden and non-permanent member of the family. In rural Bangladesh, most girls are landless. Very few girls have land under their name. A study was conducted on one village of Bangladesh that pointed out that among 40 women, only four received their share of inheritance. In addition, in the name of local customs and traditional culture, women are discriminated in their actual right and its practice (Sultana 2010).

Women’s position and status are formed around a series of cultural and economic factors such as resource access and use, ownership, control, legal and ideological structures, education, and information (Parveen 2007). Many Muslim women receive no share of their inheritance. Some are forced by their families to turn their inheritance over to their brothers. Worse yet, many brothers take the inheritance and disappear from the lives of their sisters who have no closer male relative obligated to support them or capable of doing so (Al-Hibri 2000–2002). It is a matter of shame, particularly in rural society for them to ask for the property unless they are offered (Social Institutions & Gender Index 2014). They are considered inferior in the family and society which prevents them from access to ownership right and control of their property.

There is strong evidence that financial resources under the male members of society and women often know their paternal property in details. For this reason, women often can access their property (Sarwar et al. 2007). After marriage, females normally leave their father’s house and live with their husbands. Most of the time, brothers hide important documents about the property and conceal their sisters’ entitlements. In most cases, women cannot claim their properties against their brothers due to lack of relevant information and official hazard, and most of them are not aware of their inheritance laws. Socio-cultural roles and traditions also have negative attitudes toward females. If a woman takes her property, her brother’s relationship with her sours and she is abused by society. Hence, the majority of women are not interested in taking their property (Sourav 2015).

It is thornier for sisters to file litigation against their brothers and face administrative and official hazards. Brothers often transfer the share of land that belongs to the sister without informing them, and women are left oblivious about their inherited immovable property. Most times, women do not know about the parental and husband’s property. They do not maintain information and particulars of the property. In addition, most of them are not aware of any land related laws, their right to inheritance, and have no documents as a claimant. Usually, women and poor are dropped from the survey record and the succession of the inheritance (Sourav 2012). The table below compares the current social system of women property rights and Islamic law (Table 3).

**Table 3:** Comparison between current social practice and Islamic law

Subject	Islamic property distribution system	Current social practice
Financial independence	Woman has financial independence	Woman does not have financial independence
Property inheritance	Woman has fixed shares in the inheritance property	Woman is deprived of the inheritance property
Maintenance for family	Man is responsible for providing maintenance for women and children from his property	There is no rule regarding this. In many cases, man takes over woman’s property to provide maintenance for the family
Bridal gift	It is compulsory on man to provide a bridal gift to woman	Woman/her family is required to provide bridal gift to man on many occasions

## Discussion on women’s property sharing in Bangladeshi society and the role of the *Faraid*

In Bangladeshi society, women have almost lost their right to property as they do not have any access to property ownership. They have no financial independence (Mohajan 2012). We have mentioned here two real cases recorded in the court regarding the dower (Mahar) which is also the property right of women in Islam. Case No. 1, High Court Division (Civil Revisional Jurisdiction), Abdul Rahman (Petitioner) VS Shahanara Begum (Opposite Party), Judges Fazle Hussain, Mohammad Habibur Rahma J. and Kazi Ebadul Hoque J. Date of Judgment August 8, 1990 (Monsoor 2005, p. 259). Case No. 2, High Court Division (Civil Revisional Jurisdiction), Atiqul Huque Chowhury (Petitioner) VS Shahana Rahim and anothers (Opposite Parties), Judges Mahmudul Amin Chowdhury J. and KM Hasan J. Date of Judgment February 20, 1995 (Monsoor 2005, p. 259). The previous chapter discussed how cultural values and social norms discriminate against women from getting their inheritance property. Because of the strong cultural norms in rural Bangladesh, the majority of women are landless. Although a few women own land, they have very little right to control and use their properties. Women are sometimes forced to leave their properties to their brothers. Finally, very few women are able to take legal action as they lack education. Therefore, we can put the features of discrimination on women as follows:

- Most women are completely deprived of their inheritance property.
- Those who own property lack the power to control and use the property (Sultana 2010).
- Cultural norms force a woman to leave her inheritance to her brothers.
- Those who ask for inheritance property from their brothers are considered odd in the culture and are made to suffer mentally and emotionally.
- In reality, very few women are able to ask for legal action to get their inheritance (Al-Hibri 2000–2002).

Because of this discrimination, women are financially dependent on men and are considered a burden on the society. They receive less education compared with their male counterparts and are regarded as a inferior gender (Parveen 2007). If this practice continues then, it will be a barrier for the country to develop as women represent nearly half of the population. This means that property sharing in Bangladesh should be changed.

As discussed earlier, women are discriminated against in the social practice. Only four out of 40 women receive their inheritance property (Sultana 2010) with most not receiving any share in the property (Al-Hibri 2000–2002). In contrast, the Islamic model has a fixed portion of property for women (Fowzi 1983). Though a daughter gets half of the son, there is always a share for her. Under no circumstances is the son allowed to take the daughter’s property. Therefore, we can conclude that while social model does not confirm a certain share for women, the Islamic property law always allocates a certain share for women.

Islam has prescribed its *faraid* law based on the roles and responsibilities of man and woman (Ibn Kashir 1999, vol. 2, p. 292). In the Islamic social system, man alone has the following financial responsibilities:

Firstly, man has an obligation to pay the *mahar*. *Mahar* is an amount of money the man has to pay to the wife at the time of marriage (Jassas 1984, vol. 3, p. 149). The amount of *mahar* is mutually agreed between the husband and wife before the marriage contract. The wife has the sole ownership of *mahar* and right to spend it according to her wish (Basha 2006, vol. 1, p. 189). There is no maximum limit for the amount of *mahar.* It depends on the will of the woman to decide the amount she wants. Allah says, “And give the women (on marriage) their dower as a free gift; but if they, of their own good pleasure, remit any part of it to you, take it and enjoy it with right good cheer” (Quran, 4: 4).

Secondly, man has the responsibility to provide *nafaqah* to his wife and children (Al-Wahidi 1994, vol. 1, p. 262). *Nafaqah* is the material support that man has to provide to his wife and children. *Nafaqah* includes the fundamental elements for sustenance which are food, clothes, and house (Shafi’ 2002, vol. 5, p. 127). In Islam, it is compulsory for the husband to provide *nafaqah* to the family while it is not compulsory for a wife to spend for children (Quran, 1: 233). However, women can voluntarily spend money for the family, but husbands cannot force their wives to do this. Allah says, “Let the man of means spend according to His means: and the man whose resources are restricted, let Him spend according to what Allah has given Him. Allah puts no burden on any person beyond what He has given Him. After a difficulty, Allah will soon grant relief” (Quran, 4: 34, 65: 7). Furthermore, a husband should bear the expenses of marriage ceremonies e.g. *walimah* (a ceremony soon after marriage). In this context, the following prophetic narration can be referred to. The Prophet Muhammad (PBUH) advised Abdur Rahman bin Auf to arrange a *walimah* after his marriage saying that ‘make *walimah* thought by sheep’ (Al-Bukhari 2001, vol. 7, p. 24, Hadith no. 5167). A man is also responsible for providing financial support to the close relatives i.e. mother and sister.

Regarding the just distribution in the *faraid* system, Sayed Qutub mentioned that a man’s financial responsibility is always double that of a woman. He stated:

There is no question here of favouring one sex over another. It is all a matter of maintaining balance and justice between the responsibilities of a male and those of a female within the family. In the Islamic social system, the husband is required to support his wife. He is further required to support all his children in all situations, whether he remains married to his wife or he divorces her. A woman, on the other hand, may be required to look after herself, or she may be looked after by a man both before and after her marriage. Under no circumstances is she required to maintain her husband or her children. This means that a man shoulders at least double the burden of a woman within the family and in the Islamic social system. This is how justice is maintained in this wise distribution which achieves the perfect balance between rights and duties, claims and liabilities (Qutub 2011, vol. 3, p. 42).

Based on this, the male’s share in the property is like gross income and they have to deduct a portion of his property to pay for his wife and family as well as the *mahar*. On the other hand, the female’s share is similar to net income as she is not entitled to pay anything for her husband and family. If a woman is married, then she is not responsible for her maintenance. Therefore, her share is kept for her financial security only in case she divorces her husband (Al-Hibri 2000–2002).

In the Islamic inheritance distribution system, a man does not always get a double or a higher share than the woman. There are many cases where a woman gets the same or more than a man (Al-Dawlibi 1983). If the deceased person left a daughter, father, and mother, then the daughter will get half the property. The mother will get one-sixth, and the father will get the rest. In this case, the daughter gets more than the father who is a man.

Along with this, the mother and father get equal shares when the deceased has left a son and daughter or two daughters (Quran, 4:11). Moreover, if the deceased left brothers and sisters then they equally share one-third of the property (Quran, 4:12). Also, when a woman dies, and she leaves her husband and sister, then each of them will get the half of the property.

In Bangladeshi society, there is no clear and specific share for a woman based on the relationship with the deceased. Only some close relatives like sons, daughters, wives, and sisters share in the property. However, the Islamic property distribution law is comprehensive where the share of property for a number of relatives at different stages has been discussed (Al-Wahidi 1994, vol. 1, pp. 254–255). The Islamic property law shows the property sharing system among sons, daughters, granddaughters, grandsons, brothers, sisters, step brothers, step sisters, fathers, mothers, uncles, etc. Along with this, it prescribes the law of inheritance for different cases of relatives, for example, when there are only one daughter and father then the property distribution is different (Quran, 4: 11).

If we look into the root of the social practice of property distribution in Bangladesh, we notice that it results from the misunderstanding and misrepresentation of the Islamic system (Mohammad 2013). The inheritance property was distributed among the Muslims in Bangladesh based on Islamic law (Kamal 2015). However, over the course of time people violated the Islamic laws. Some of them practice part of Islamic law of property distribution. Eventually, it became a culture among the people of Bangladesh (Bulbul 2013) leading some to believe that the social practice of property sharing is the Islamic law. Based on this, some people urged changing the Islamic property sharing law. However, the Islamic property sharing system is far different from the social practice in Bangladesh. The Islamic property law is based on fairness and justice while the social model is based on injustice between male and female (Mohammad 2013).

The Islamic law of property sharing will make women economically more solvent. They will be more secure and independent financially. When a woman has a certain share in the property, then they will be less dependent on their husbands and fathers. Women will be financially solvent as they have a share in the property (Al-Owzah 1994). Women are not obliged to spend money for the family (Ibn Abbus, n.d, p. 69.). Therefore, her property will remain her financial security in case of divorce (Fatima 2009).

If the *faraid* is practised, then there will be a balance between males and females in the society (Mohammad 2013). This is because women will be richer in the society as they have property. Economic independence will empower Bangladeshi women. Consequently, they will take a more active part in the society. They will have more opportunity to get a higher education, and they will be able to get more jobs. Women will rid themselves of their unfortunate status of an inferior gender in the society as they are not burdensome on a man (Abdullah et al. 2015).

Sometimes, because of non-property ownership, woman become involved in immoral activities. The Islamic law of property sharing will improve morality by ensuring their property rights. On the other hand, their religious belief and confidence will develop as the religion has given them financial solvency and they will be more respectful to their religion (Mufty 2010, p. 9). Therefore, the government of Bangladesh should continue with the Islamic law of property sharing.

## Conclusion

Based on the discussion on two property sharing systems above, the study concludes that the Islamic property distribution system is able to bring about justice between man and woman in property sharing. Islamic system may solve the discrimination against women in property. Therefore, it is not recommended to legislate a new property sharing law in Bangladesh. Rather, it is highly recommended to enforce the Islamic system among the people. If the Islamic law is implemented, a woman will be more secure economically. Moreover, it will help the government to empower women because the Islamic law renders women more independent and powerful in the society. Consequently, they will not be marginalised in the society. However, there are some challenges in implementing the *Faraid* system in Bangladesh. Firstly, many people oppose the *Faraid* system due to a lack of proper knowledge about Islamic law in property sharing. Secondly, many rapacious men who want to possess women’s property disagree with the Islamic system. Thirdly, some are not interested in practicing Islamic law in property distribution. Others are not aware of the property right.

Steps are needed to overcome the problems. People should be educated about the Islamic law in property distribution. The government should create the opportunity for the women to secure their rights in property. Lack of interest and awareness can be resolved by increasing public interest and create awareness through mass media among the people. Scholarly articles also are needed to address the problems. Finally, the enforcement of the Islamic inheritance law by the government is recommended. Islamic scholars should help the government identify the positive side of the Islamic property sharing system.
